# A Very Low Geno2pheno False Positive Rate Is Associated with Poor Viro-Immunological Response in Drug-Naïve Patients Starting a First-Line HAART

**DOI:** 10.1371/journal.pone.0105853

**Published:** 2014-08-25

**Authors:** Daniele Armenia, Cathia Soulie, Domenico Di Carlo, Lavinia Fabeni, Caterina Gori, Federica Forbici, Valentina Svicher, Ada Bertoli, Loredana Sarmati, Massimo Giuliani, Alessandra Latini, Evangelo Boumis, Mauro Zaccarelli, Rita Bellagamba, Massimo Andreoni, Anne-Geneviève Marcelin, Vincent Calvez, Andrea Antinori, Francesca Ceccherini-Silberstein, Carlo-Federico Perno, Maria Mercedes Santoro

**Affiliations:** 1 Department of Experimental Medicine and Surgery, University of Rome Tor Vergata, Rome, Italy; 2 Unité Mixte de Recherche en Santé (UMR_S) 1136 Pierre Louis Institute of Epidemiology and Public Health, Université Pierre et Marie Curie (UPMC) University Paris 06, Paris, France; 3 UMR_S 1136 Pierre Louis Institute of Epidemiology and Public Health, Institut National de la Santé et de la Recherche Médicale (INSERM), Paris, France; 4 Laboratoire de Virologie, Assistance Publique-Hôpitaux de Paris (AP-HP), Groupe hospitalier Pitié Salpêtrière, Paris, France; 5 Antiviral Drug Monitoring Unit, Istituto Nazionale delle Malattie Infettive (INMI) Lazzaro Spallanzani, Rome, Italy; 6 Molecular Virology, University Hospital Tor Vergata, Rome, Italy; 7 Infectious Disease Unit, University Hospital Tor Vergata, Rome, Italy; 8 Department of Infectious Dermatology, San Gallicano Hospital, Rome, Italy; 9 Infectious Diseases Division, Istituto Nazionale delle Malattie Infettive (INMI) Lazzaro Spallanzani, Rome, Italy; University of British Columbia, Canada

## Abstract

**Background:**

We previously found that a very low geno2pheno false positive rate (FPR ≤2%) defines a viral population associated with low CD4 cell count and the highest amount of X4-quasispecies. In this study, we aimed at evaluating whether FPR ≤2% might impact on the viro-immunological response in HIV-1 infected patients starting a first-line HAART.

**Methods:**

The analysis was performed on 305 HIV-1 B subtype infected drug-naïve patients who started their first-line HAART. Baseline FPR (%) values were stratified according to the following ranges: ≤2; 2–5; 5–10; 10–20; 20–60; >60. The impact of genotypically-inferred tropism on the time to achieve immunological reconstitution (a CD4 cell count gain from HAART initiation ≥150 cells/mm^3^) and on the time to achieve virological success (the first HIV-RNA measurement <50 copies/mL from HAART initiation) was evaluated by survival analyses.

**Results:**

Overall, at therapy start, 27% of patients had FPR ≤10 (6%, FPR ≤2; 7%, FPR 2–5; 14%, FPR 5–10). By 12 months of therapy the rate of immunological reconstitution was overall 75.5%, and it was significantly lower for FPR ≤2 (54.1%) in comparison to other FPR ranks (78.8%, FPR 2–5; 77.5%, FPR 5–10; 71.7%, FPR 10–20; 81.8%, FPR 20–60; 75.1%, FPR >60; p = 0.008). The overall proportion of patients achieving virological success was 95.5% by 12 months of therapy. Multivariable Cox analyses showed that patients having pre-HAART FPR ≤2% had a significant lower relative adjusted hazard [95% C.I.] both to achieve immunological reconstitution (0.37 [0.20–0.71], p = 0.003) and to achieve virological success (0.50 [0.26–0.94], p = 0.031) than those with pre-HAART FPR >60%.

**Conclusions:**

Beyond the genotypically-inferred tropism determination, FPR ≤2% predicts both a poor immunological reconstitution and a lower virological response in drug-naïve patients who started their first-line therapy. This parameter could be useful to identify patients potentially with less chance of achieving adequate immunological reconstitution and virological undetectability.

## Introduction

Despite the great progress in treating HIV-1 infection, in some patients starting their first treatment the effectiveness of highly active antiretroviral therapy (HAART) is still not sufficient, with consequent virological failures [1]–[4]. Furthermore, although antiretroviral therapy improves immune response, some patients infected with human immunodeficiency virus type 1 (HIV-1) present unsatisfactory CD4 T cell recovery despite achieving viral suppression, resulting in increased morbidity and mortality [4]–[10]. In this regard, an increase in CD4 cell count in the range of 50 to 150 cells/mm^3^ per year (generally with an accelerated response in the first 3 months of treatment) is considered an adequate CD4 response for most patients starting their first-line regimen [11]–[12]. It has been shown that the use of the CXCR4 co-receptor is generally seen in more advanced stages of disease, and has been associated with an increased severity of HIV disease, higher viral load, and a decreased CD4 cell count [13]–[16]. In the absence of antiretroviral therapy, CXCR4-using viruses (X4), detected by phenotypic or genotypic assays, are associated with faster CD4 cell count decreases, regardless of baseline CD4 cell count or viral load [14], [15], [17]–[19]. However, in the presence of antiretroviral therapy, this issue has still been poorly investigated, and the available results are controversial [20]–[23].

Nowadays, especially after the introduction of CCR5-antagonists in clinics, the determination of HIV-1 tropism is beginning to be routinely performed. For this reason, the classical phenotypic assays, such as Trofile [24], are taken over by more cost-effective genotypic tests in the large majority of countries.

One of the most widely used tools for tropism determination is geno2pheno [coreceptor] (G2P) [25]. By using the genetic information contained in the sequence of HIV-1 gp120 V3-loop, this web-tool gives a percentage score (false positive rate [FPR]) that allows us to estimate the probability of having CCR5-using virus (R5). G2P has been shown to have good concordance with classical phenotypic tests [26]. So far, the FPR cut-off of 10% is recommended by European guidelines to discriminate R5- and X4-infected patients using G2P system [27]. However, there is evidence indicating that this system can provide reliable discrimination between R5 and X4 sequences even when FPR is set at lower values [26], [28], [29].

Notably, beyond the crude tropism determination, some recent studies (including ours) provided new important information about the relevance of FPR in terms of association with viro-immulogical parameters and X4-tropic intra-patient quasispecies prevalence. Indeed, by a cross-sectional study we demonstrated that within the context of genotypically-inferred CXCR4 tropism, the FPR ≤2% defines (far better than 10%-FPR) a viral population associated with low CD4 count, with potentially greater cytopathic effect, and with more advanced disease both in HAART-naïve and HAART-experienced patients [30]. Moreover, we found that FPR by V3 population sequencing can predict the burden of HIV-1 CXCR4-using species detected by 454-pyrosequencing. In particular, at very low FPR (≤2%) by population sequencing the highest prevalence of X4-species by ultra-deep pyrosequencing was observed [31].

In view of all these considerations, the aim of this longitudinal study was to evaluate whether FPR ≤2% at the moment of starting HAART might be associated with viro-immunological responses of the first-line regimen.

## Materials and Methods

### Patients

HIV-1 infected patients starting a first-line regimen not including CCR5-antagonists in several clinical centres from Italy and France were selected on the basis of the following criteria: i) B subtype infected; ii) age ≥18 years; iii) V3-genotyping test available at therapy starting (in the time-window from 6 months before to the moment of therapy initiation); iv) pre-HAART CD4 cell count and viral load measurements available in the time-window from 3 months before to 1 week after HAART initiation; v) at least one CD4 cell count and viral load measurement available after 6 months of therapy. In patients with more than 1 viral load or CD4 cell count before HAART started, the last measurement was considered as pre-HAART value.

### Ethics statement

Approval by Ethics Committee was deemed unnecessary because, under Italian law, such an approval is required only in the hypothesis of clinical trials on medicinal products for clinical use (art. 6 and art. 9, leg. decree 211/2003). This research was conducted on [samples and] data already available, and not collected for this study. CD4 cell counts were previously determined for each patient only for clinical reasons and not for research. All samples and data used in the study were previously anonymized, according to the requirements set by Italian Data Protection Code (leg. decree 196/2003) and by the General authorizations issued by the Data Protection Authority.

### CD4 cell count and HIV-RNA quantification

Flow cytometry from whole blood was used to determine CD4 cell counts at each study visit. Depending on methodologies available at the different clinical centers participating in this study, plasma viremia was determined using three different assays: the Roche Cobas CA/CTM version 2.0 (Mannheim, Germany), the Abbott RealTime HIV-1 (Chicago, Illinois), and the VERSANT HIV-1 Version 3.0 (Bayer Corporation, Diagnostics Division, Tarrytown, New York) [32], [33]. Previous studies demonstrated that even if there was not a uniform approach regarding the HIV-1 viral load detection, the results obtained by these assays correlated very well, only a few samples having a difference of more than 0.5 log_10_ copies/mL [34], [35]. For 304/305 patients (99.7%) viremia measurements were quantifiable above 500,000 copies/mL.

### Genotyping

Sequencing of HIV-1 *pol* gene (containing the entire protease and the first 240/335 amino acids of the reverse transcriptase open reading frame) and of HIV-1 gp120 V3-loop was performed using plasma samples collected from the patients before their first-line therapy. For about 90% (N = 274) of plasma samples, *pol* genotypic tests used in this analysis were performed by means of a commercially available kit (The ViroSeq HIV-1 Genotyping System, Abbott Molecular, Des Plains, Illinois, USA) according to the manufacturer's recommendations [36]. HIV-1 gp120 V3 loop sequencing for these 274 samples was performed by using a well validated diagnostic-use protocol, based on commercially available RNA-extraction (QIAamp RNA Viral Mini kit, Qiagen), reverse-transcription and amplification (SuperScript One-Step RT-PCR for Long Templates – Invitrogen) and genotyping (BigDye terminator version 3.1 cycle sequencing kit, Applied-Biosystems, Foster City, CA) kits [37]. Amplified gp120 V3 products were full-length sequenced in sense and antisense orientations by an automated sequencer (ABI 3130 XL) by using four different overlapping sequence-specific primers to ensure the coverage of the V3 sequence by at least two sequence segments [37].

For the remaining 31 plasma samples, HIV-1 *pol* and gp120 V3 loop sequencing were performed by using the technique of ANRS (French National Agency for AIDS Research, described on the web site HIV French resistance: http://www.hivfrenchresistance.org/) [38].

To estimate the prevalence of transmitted drug resistance at the start of HAART, the list of mutations reported by Bennett *et al*., 2009 [39] was used. Subtype has been determined by using the phylogenetic approach, as previously described [40]. The genotypic susceptibility score (GSS) for optimized therapy was also calculated according to Rega algorithm (version: v8.0.2; http://regaweb.med.kuleuven.be/software/rega_algorithm/), based on the sum of genotype sensitivities to all drugs prescribed in the HAART. GSS for single drugs was scored as 0 (resistant virus), 0.5 (virus with intermediate resistance) and 1 (susceptible virus).

### Genotypic prediction of viral tropism

HIV-1 co-receptor usage was determined from the V3 nucleotide sequence by using the G2P algorithm available at the following website http://coreceptor.bioinf.mpi-inf.mpg.de/ [41]. G2P was set at FPR of 10%, as recommended by current guidelines [27]. To evaluate the impact of the burden HIV-1 CXCR4-using species on immunological and virological response, FPR values were further stratified according to the following 6 FPR (%) ranges: ≤2; 2–5; 5–10; 10–20; 20–60; >60, as previously described [31]. In this categorization all 6 ranges are left-open and right-closed (e.g. ≤2; >2 and ≤5; >5 and ≤10; >10 and ≤20; >20 and ≤60; >60).

### Statistical analysis

All the analyses were performed using the statistical R open source environment (version 3.0.2) and the software package SPSS (version 19.0) for Windows (SPSS Inc., Chicago, Illinois).

### Survival analyses

To estimate the time to achieve immunological reconstitution and viral load undetectability, Kaplan-Meier curves were used. Log-rank test for trend was implemented for FPR values stratified. To estimate the predictive impact of genotypically-inferred tropism on immunological reconstitution and virological success, Cox proportional hazard models were used. Immunological reconstitution was defined as a CD4 cell count gain from HAART initiation of at least 150 cells/mm^3^ [11]–[12]. Virological success was defined as the time of the first HIV-RNA measurement <50 copies/mL from HAART initiation as the most common undetectability cut-off currently considered for HIV-RNA [11]–[12], [32].

Cox's analysis was performed, evaluating the proportional hazards assumption, on the full set of patients, regardless of therapy changes. Patients that interrupted therapy for any reason were censored at the moment of the treatment stopping. In the multivariable Cox proportional hazard model, the variables used as potential confounders were: FPR (set at 10% and/or FPR [%] stratified for the following ranks: ≤2; 2–5; 5–10; 10–20; 20–60; >60), age, gender, risk factor, pre-HAART CD4 cell count, pre-HAART viremia, hepatitis C coinfection, year of starting treatment, transmitted drug resistance, third drug used (non-nucleoside reverse transcriptase inhibitor [NNRTI] vs. ritonavir-boosted protease inhibitor [PI/r] vs. raltegravir), nucleos(t)ide reverse transcriptase inhibitor (NRTI) backbone used, number of drugs being used (≤3 vs. >3 drugs).

Since the prevalence of X4- or dual/mixed-tropic variants tends to be increased by decreasing CD4 cell count levels [42], [43], CD4 cell counts at baseline were stratified into the following ranges: ≤50, 51–100, 101–200, 201–350 and >350 cells/mm^3^.

Pre-HAART viremia values were stratified in the following ranks: ≤30,000; 30,001–100,000; 100,001–300,000; 300,001–500,000; 500,001–1,000,000 and >1,000,000 copies/mL.

## Results

### Patients' characteristics at HAART initiation

Overall, 305 patients satisfying all criteria were included in the present analysis. Baseline characteristics are summarized in Table 1. The prevalent mode of transmission was the homosexual route (107, 35.1%). 77.7% of patients had CD4 cell count <350 cells/mm^3^. About half of patients showed HIV-1 viral load >100,000 copies/mL; in particular, 10.5% and 6.6% of patients had viremia 500,001–1,000,000 and >1,000,000 copies/mL, respectively. Twenty-four (7.9%) patients were coinfected with hepatitis C virus.

**Table 1:** Characteristics of 305 HIV-1 B subtype infected patients starting their first-line HAART.

Characteristics	Categories	Overall (N = 305)
**Gender, n (%)**	Male	252 (82.6)
**Age, Median (IQR)**	Years	41 (34–46)
**Pre-HAART CD4 (cells/mm^3^), n (%)**	≤50	48 (15.7)
	51–100	24 (7.9)
	101–200	48 (15.7)
	201–350	117 (38.4)
	>350	68 (22.3)
**CDC stage, n (%)**	A	107 (35.1)
	B	91 (29.8)
	C	68 (22.3)
	Unknown	39 (12.8)
**Pre-HAART HIV-RNA (copies/mL)^a^, n (%)**	≤30,000	82 (26.9)
	30,001–100,000	66 (21.6)
	100,001–300,000	82 (26.9)
	300,001–500,000	23 (7.5)
	500,001–1,000,000	32 (10.5)
	>1,000,000	20 (6.6)
**Genotypic determination of HIV tropism^b^, n (%)**	X4	84 (27.5)
	R5	221 (72.5)
**Risk factor, n (%)**	Heterosexual	80 (26.2)
	Homosexual	107 (35.1)
	Drug addiction	15 (4.9)
	Sexual	32 (10.5)
	Other or unknown	71 (23.3)
**Coinfection, n (%)**	Hepatitis C	24 (7.9)
**Transmitted drug resistance^c^, n (%)**		24 (7.9)
**HAART initiation, Median (IQR)**	Year	2010 (2009–2011)
**NRTI backbone, n (%)**	TDF+FTC	258 (84.6)
	AZT+3TC	17 (5.6)
	Other^d^	30 (9.8)
**Third drug, n (%)**	NNRTI	112 (36.7)
	Ritonavir boosted PI	161 (52.8)
	Raltegravir^e^	32 (10.5)
**More than 3 drugs used, n (%)**		21 (6.9)
**Adherence level^f^, n (%)**	High	266 (87.2)
	Median	19 (6.2)
	Low	18 (5.9)
	Unknown	2 (0.7)
**CD4 cell count measurements, Median (IQR)**	Number per patient	6 (4–10)
**Time of CD4 cell count follow-up from starting HAART, Median (IQR)**	Months	12 (8–19)
**Viral load measurements, Median (IQR)**	Number per patient	9 (6–12)
**Time of HIV-RNA follow-up from starting HAART, Median (IQR)**	Months	19 (13–27)

a. Viremia was not quantifiable above 500,000 copies/mL only for one patient. We arbitrarily included this patient in the viremia level 500,001–1,000,000 copies/mL; b. Geno2pheno false positive rate set at 10%; c. As least 1 mutation associated with resistance to protease inhibitors, nucleos(t)ide reverse transcriptase inhibitors and/or non-nucleoside reverse transcriptase inhibitors, according to surveillance list from Bennett *et al.* 2009 [39]; d. ABC+3TC (n = 11); TDF+3TC (n = 3); DDI+3TC (n = 1); NRTI sparing (n = 15); e. Patients treated with raltegravir were considered as independent category regardless the other drugs included in the same regimen; f. Data about adherence levels were retrieved from physicians' reports. ABC: Abacavir. AZT: Zidovudine. DDI: Didanosine. FTC: Emtricitabine. IQR: Interquartile range. HAART: Highly active antiretroviral therapy. NRTI: Nucleos(t)ide reverse transcriptase inhibitor. NNRTI: Non-NRTI. PI: Protease inhibitor. TDF: Tenofovir. 3TC: Lamivudine.

Nearly all patients were treated with a modern genotype-tailored HAART, including currently recommended drugs; 93% (N = 285) of patients started their first antiretroviral regimen after 2008, and 95% (N = 290) were treated with at least 2 NRTIs (most NRTIs combination used: tenofovir + emtricitabine, 258 [84.6%]) in combination with either an NNRTI (N = 112 patients, 94.5% of them treated with efavirenz) or a ritonavir-boosted PI (N = 161 patients, mainly treated with darunavir [35.4%] or lopinavir [29.8%] or atazanavir [29.2%]). Thirty-two patients (10.5%) were treated with raltegravir, mainly administered in combination with 2 NRTIs +1 ritonavir-boosted PI (25 patients, 78%). No significant correlation between the third drug used and the different FPR levels was observed (data not shown). The majority of patients attended a high compliance level (87.2%). Transmitted drug resistance was found in around 8% of patients.

Nearly all patients (99%) have been treated with effective therapy with GSS ≥3.

### Prevalence of patients infected with X4-tropic viruses at first-line HAART start

**Figure 1:**
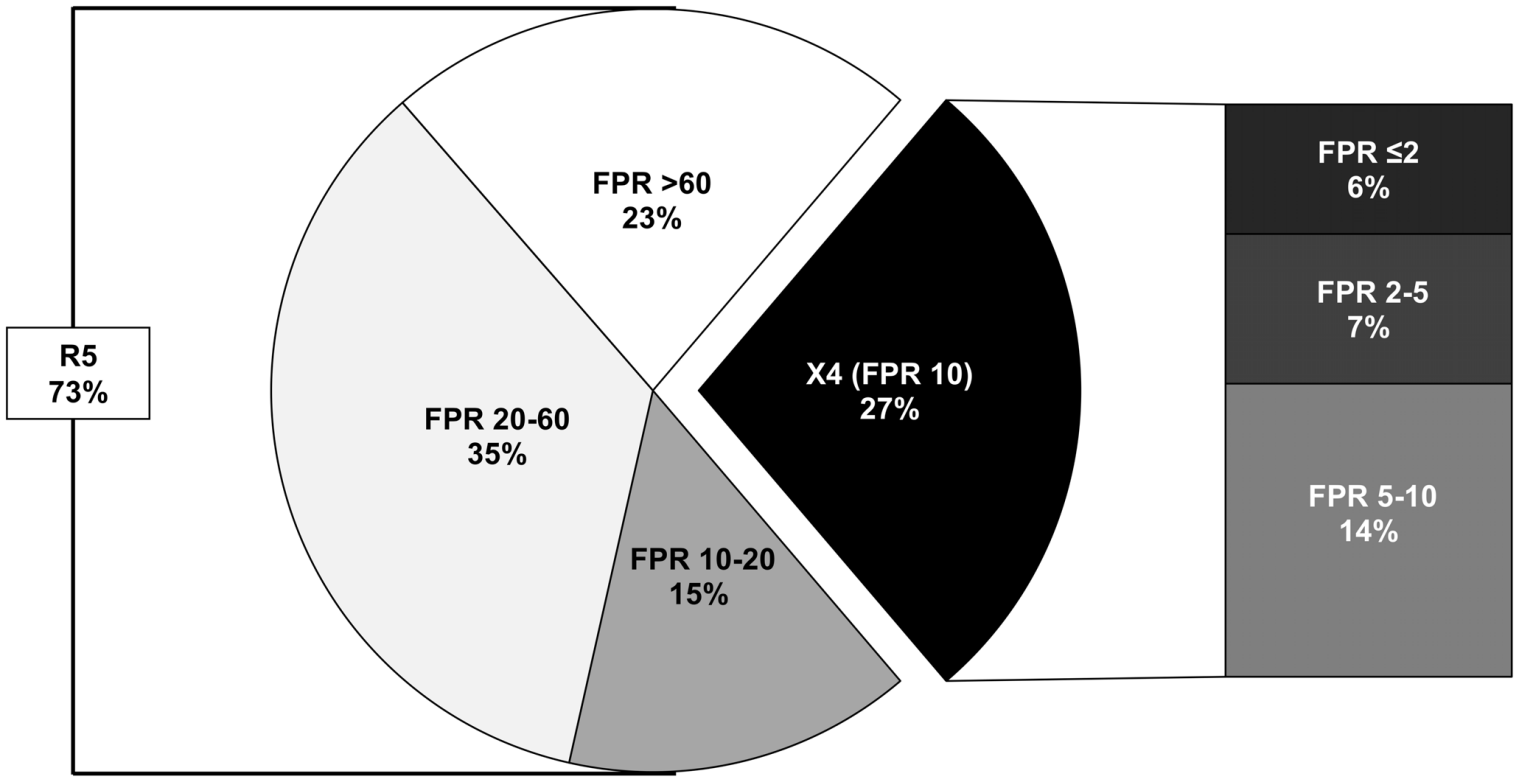
Proportion of patients infected with ×4- and R5-tropic viruses. Pie plot represents: i) the proportions of R5-infected (FPR >10%) patients according to the following FPR ranges: 10–20%, 20–60%, >60%; ii) the proportion of ×4-infected (FPR ≤10%) patients (in black). Exploded bars represent the stratification of ×4-infected patients according to the following FPR ranges: ≤2%, 2–5% and 5–10%.

V3 genotypic tropism test was performed in a median (Interquartile Range, IQR) time of 20 (5–47) days before HAART start. The proportions of patients infected with X4- or R5-tropic viruses according to different FPRs are represented in Figure 1. In particular, 82 of 305 (27%) patients showed X4-using viruses at the time of genotypic tropism testing with FPR set at 10% (FPR ≤2% = 6%; FPR 2–5% = 7%; FPR 5–10% = 14%). Among 221 patients infected by R5-tropic viruses, 31% had FPR >60% (corresponding to 23% of the overall population analyzed).

### Survival analyses for the evaluation of immunological reconstitution

In the overall population, the median time to achieve immunological reconstitution, defined as described in the Materials and Methods, was 4.4 ([95% confidence interval, C.I.]: 3.1–5.6) months. By 12 months of treatment, the probability of achieving immunological reconstitution was 75.5%.

Among patients who achieved immunological reconstitution, the 92.9% reached a CD4 cell count gain ≥150 cells/mm^3^ during the viremia drop or under virological suppression.

**Figure 2:**
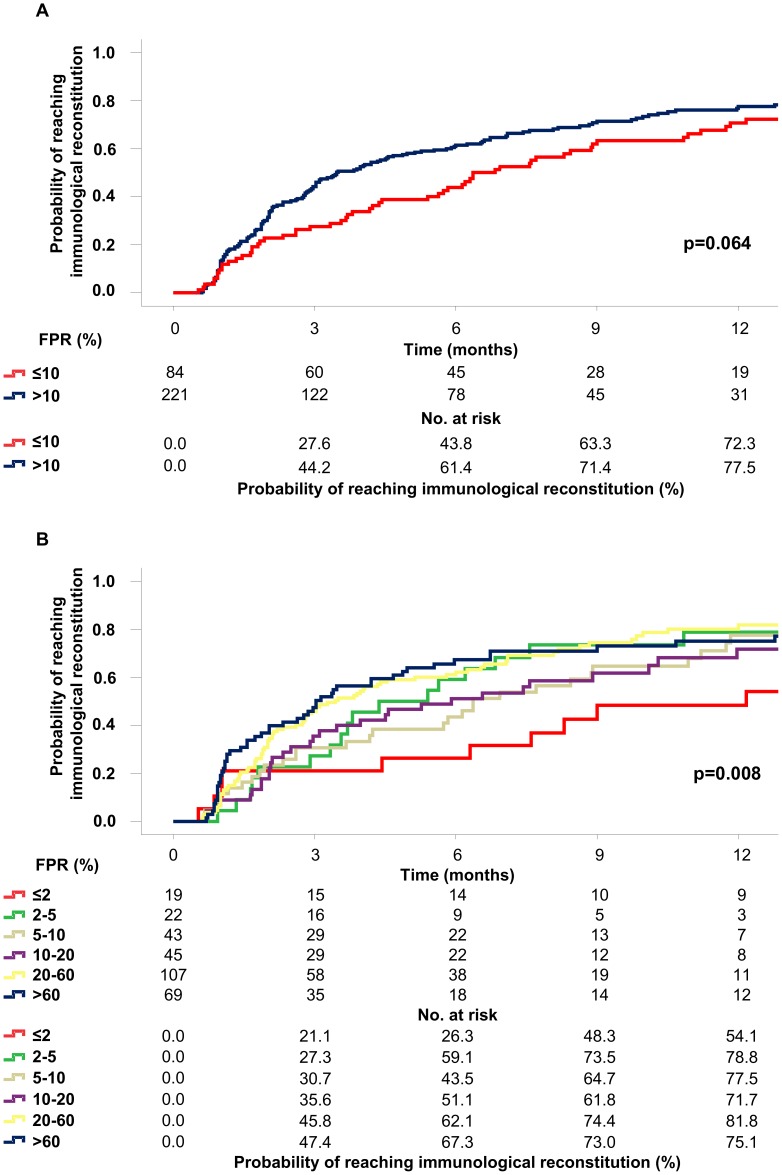
Kaplan-Meier estimates of the probability of immunological reconstitution according to HIV-1 pre-HAART genotypically-inferred tropism. The estimations of the probability of immunological reconstitution (defined as a CD4 cell count gain of at least 150 cells/mm^3^) are indicated in panels A-B. Kaplan-Meier estimation was performed considering FPR set both at 10% (panel A) and at several FPR ranges (≤2%; 2–5%; 5–10%; 10–20%; 20–60%; >60%, panel B). In each panel, the number at risk and the probability of reaching the event by 3–12 months are indicated. P values were calculated by log-rank test for trend.

Stratifying patients by using the classical 10% FPR cut-off, only a trend of difference in the rates of immunological reconstitution was observed in X4-infected patients (72.3%) compared to R5-infected patients (77.5%) (p = 0.064, Figure 2 Panel A). By contrast, by a further FPR stratification that quantitatively reflects the burden of X4 quasispecies [31], the rate of immunological reconstitution by 12 months was significantly lower for FPR ≤2% (54.1%) in comparison to other FPR [%] ranks (FPR = 2–5: 78.8%; FPR = 5–10: 77.5%; FPR = 10–20, 71.7%; FPR = 20–60, 81.8%; FPR>60: 75.1%, p = 0.008) (Figure 2, Panel B).

Both uni- and multivariable Cox models showed that the relative hazard to achieve immunological reconstitution significantly decreased in X4-infected patients with the lowest FPR rank. In particular, by univariable analysis, X4-infected patients having pre-HAART FPR ≤2% had a significantly lower relative hazard compared to R5-infected patients with pre-HAART FPR >60% (relative hazard [95% C.I.]: 0.51 [0.28–0.91], p = 0.024) (Table 2). By Cox multivariable analysis, these results were confirmed with stronger significance after adjusting for age, gender, risk factor, pre-HAART CD4 cell count, pre-HAART viremia, hepatitis C coinfection, year of starting treatment, transmitted drug resistance, third drug administered (NNRTI vs. PI/r vs. raltegravir), NRTI backbone used and an administration of more than 3 drugs (relative hazard [95% C.I.]: 0.37 [0.20–0.71], p = 0.003) (Table 2).

**Table 2:** Relative hazard to achieve viro-immunological response according to baseline FPR ranks in HIV-1 infected patients starting their first-line HAART (Cox Models).

	Relative hazard to reach immunological reconstitution				Relative hazard to reach virological success			
	(CD4 cell count gain ≥150 cell/mm^3^)				(HIV-RNA <50 copies/mL)			
FPR	Crude (95% C.I.)	P value	Adjusted^a^ (95% C.I.)	P value	Crude (95% C.I.)	P value	Adjusted^a^ (95% C.I.)	P value
>60^b^	1		1		1		1	
20–60	1.04 (0.74–1.46)	0.842	1.03 (0.70–1.50)	0.894	0.78 (0.57–1.07)	0.124	0.83 (0.59–1.16)	0.272
10–20	0.66 (0.43–1.03)	0.068	0.65 (0.41–1.03)	0.067	0.94 (0.64–1.38)	0.758	1.02 (0.68–1.53)	0.939
5–10	0.75 (0.48–1.15)	0.183	0.84 (0.52–1.34)	0.457	1.16 (0.79–1.72)	0.441	1.39 (0.92–2.12)	0.122
2–5	0.90 (0.53–1.54)	0.703	0.90 (0.50–1.61)	0.721	0.92 (0.55–1.53)	0.741	1.10 (0.65–1.87)	0.723
**≤2**	**0.51 (0.28**–**0.91)**	**0.024**	**0.37 (0.20**–**0.71)**	**0.003**	**0.51 (0.29**–**0.90)**	**0.019**	**0.50 (0.26**–**0.94)**	**0.031**

a. Adjusted for: age, gender, risk factor, pre-HAART CD4 cell count, pre-HAART HIV-RNA, hepatitis C coinfection, year of starting treatment, transmitted drug resistance, third drug used (non-nucleoside reverse transcriptase inhibitor vs. boosted protease inhibitor vs. raltegravir), NRTI backbone used, number of drug administered (≤3 vs. >3 drugs); b. Reference group (dummy); Boldface indicates the geno2pheno false positive rate (FPR) ranks that were significantly associated (p<0.05) with viro-immunological response. C.I.: confidence interval.

Repeating all the survival analyses by excluding patients with FPR ≤2%, all the associations between genotypically-inferred tropism and immunological response were lost (data non shown).

Regarding the other potential confounders evaluated, all the results are summarized in Table S1. In particular, by multivariable Cox regression analysis, as expected, patients with CD4 cell count >50 cells/mm^3^ had a higher hazard to achieve immunological reconstitution compared with those with CD4 cell count ≤50 cells/mm^3^. Among the other confounders, patients taking ziduvudine + lamivudine had a lower hazard to achieve immunological reconstitution compared to those taking tenofovir + emtricitabine. Very high pre-HAART viremia (>1,000,000 copies/mL) was significantly associated with a higher hazard to reach immunological reconstitution.

### Survival analyses for the evaluation of virological success

By Kaplan-Meier estimates, overall the median time (95% C.I.) to achieve virological success was 4.0 (3.7–4.5) months. The overall probability of achieving virological success was 95.5% at 12 months. No difference in terms of rates of virological suppression was observed between X4- and R5-infected patients regardless of FPR levels (data not shown). However, by univariable Cox analysis, patients with FPR ≤2% showed a significantly lower hazard to achieve virological success compared to patients with FPR >60% (relative hazard [95% C.I]: 0.51 (0.29–0.90); p = 0.019) (Table 2). By multivariable analysis, adjusting for age, gender, risk factor, pre-HAART CD4 cell count, pre-HAART viremia, hepatitis C coinfection, year of starting treatment, transmitted drug resistance, third drug administered (NNRTI vs. PI/r vs. raltegravir), NRTI backbone used and more than 3 drugs administered, this result was confirmed (relative hazard [95% C.I]: 0.50 (0.26–0.94); p = 0.031) (Table 2).

Repeating all the analyses by excluding patients with FPR ≤2%, all the associations between genotypically-inferred assessed tropism and virological response were lost (data non shown).

Among the other confounders, very high pre-HAART viremia (>500,000 copies/mL) was significantly associated with poorer virological response, as previously observed [32] (Table S1). Of note, the lowest hazard of virological undetectability was found in patients with pre-HAART viremia >1,000,000 copies/mL. The use of raltegravir was associated with a higher hazard to achieve virological success compared with an NNRTI or a PI/r based regimen.

## Discussion

By this longitudinal study we found that FPR ≤2% is an independent predictor of both a poor immunological reconstitution and a lower virological response in HIV-1 B subtype infected patients who have initiated their first-line antiretroviral regimen. Repeating our analyses by excluding patients with FPR ≤2%, all the associations between genotypically-inferred tropism and viro-immunological response were lost, confirming the crucial role of FPR ≤2%.

These results reinforce our hypothesis that the highest intra-patient prevalence of X4 variants is found in patients with very low FPRs [30], [31], and that, in this particular situation, high prevalence of X4-species might influence the CD4 recovery after therapy start. Furthermore, it was recently observed that failures of maraviroc-containing regimens select only viruses with an extremely low FPR, implying that FPR ≤2% could indicate the presence of pure-X4, really insensitive to anti-CCR5 antagonists [44].

The analysis described in the results section refers to a time frame of 12 months. By extending our analysis to 36 months of treatment, more than 90% of patients achieved a gain of CD4 cell count ≥150 cells/mm^3^ (data not shown). Also in this case, patients with FPR ≤2% showed the lowest probability (83.4%) compared to those having higher FPR ranks. Thus, these findings suggest that the negative effect of a very low FPR is maintained in the long term in patients starting first HAART, and its predictive value could be relevant to identifying patients with a blunted increase in their CD4 cell counts. *Ad hoc* studies to clarify the role of tropism in long term suppressed HIV-1 patients are needed to confirm these results.

Findings about immunological reconstitution are reported in the ArTEN and ANRS 130 APOLLO studies: genotypically-assessed viral tropism (with FPR set at 10% and 5.75%) did not seem to impact on the extent of CD4 cell count recovery on antiretroviral therapy [22], [23]. These results are in line with our observations, showing that only FPR ≤2% is related to immune reconstitution, while FPR values >2 are not associated with this parameter [23].

Our data also show a relationship between FPR ≤2% and virological response. Indeed, by both uni-and multivariable Cox analyses we found a lower hazard to achieve virological suppression for this FPR range if compared with the others, suggesting that patients carrying a pure X4 tropic virus are potentially in a compromised immunological status and consequently might have a lower and/or delayed virological suppression after first-line HAART. These findings are in agreement with the results obtained in the ArTEN study in which HIV-1 B subtype X4-infected patients showed a lower virologic response compared to those R5-infected at 48 weeks from their first-line HAART [22].

This study may have some limitations. First, tropism was inferred by the analysis of only V3 sequences. Indeed, it is known that other residues outside of V3 loop within gp120 and gp41 could be relevant for viral coreceptor usage [45]–[47]. In addition, our cohort includes only B subtype infected patients. While this has been done on purpose to perform cleaner analyses, at the same time the results cannot be extrapolated to non-B infected patients. The accuracy of genotypic tools to assess viral tropism is reported to be so far lower with non-B subtypes than with clade B variants [16], [48], [49], therefore any extrapolation to non-B subtypes must be done with great caution.

Furthermore, we did not evaluate the potential role of therapy compliance on viro-immunological response. It is known that less adherent patients have a higher risk of death and of inadequate CD4+ count recovery [50]. In this study we did not find any correlation between adherence level and immunological recovery (data not shown). On the other hand, as expected in a population achieving a high rate of virological success, around 90% of patients attended high compliance to therapy regardless FPR levels; thus we decided not to consider adherence level as potential confounder in the Cox analyses. Moreover, we decided not to take account of the reason/timing of potential therapy switches during the observation of patients. Indeed, only 5% of patients changed therapy before the achievement of virological success. These few patients changed their regimen within 3 months showing a similar rate of immunological reconstitution compared to those who never changed therapy (data not shown).

Finally, it would be interesting to evaluate the relationship between immunological reconstitution and genotypically-inferred tropism in patients with acute infection. However, this category of patients could not be assessed in this analysis, since in clinical practice the diagnosis is frequently made after a time of infection that cannot be quantified.

In conclusion, our findings show that FPR ≤2% defines patients carrying a viral population significantly associated with both poorer immunological reconstitution and lower and/or delayed virological response in HIV-1 B infected patients starting their first-line therapy. These data reinforce a previous suggestion that FPR ≤2% may not only identify those patients whose virus is insensitive to CCR5-inhibitors, but can also be useful to identify patients potentially with less chance of achieving adequate viro-immunological response.

## Supporting Information

Table S1 **Factors related to immunological and virological response in HIV-1 infected patients starting their first-line HAART (Cox Models).**
(DOC)
