# Supplementary material for: A Very Low Geno2pheno False Positive Rate Is Associated with Poor Viro-Immunological Response in Drug-Naïve Patients Starting a First-Line HAART
Source: PLoS One. 2014 Aug 25;9(8):e105853. doi: 10.1371/journal.pone.0105853 (PMC4143365; doi:10.1371/journal.pone.0105853)
Supplement: Table S1 — Factors related to immunological and virological response in HIV-1 infected patients starting their first-line HAART (Cox Models). (DOC) [file pone.0105853.s001.doc]

| **Table S1. Factors related to immunological and virological response in HIV-1 infected patients starting their first-line HAART (Cox Models).** | | | | | | | | | |  |
| --- | --- | --- | --- | --- | --- | --- | --- | --- | --- | --- |
|  |  | **Probability of reaching immunological reconstitution** | | | |  | **Probability of reaching virological success** | | | |
| **Variables** |  | **(CD4 cell count gain >150 cell/mm^3^)** | | | |  | **(HIV-RNA <50 copies/mL)** | | | |
|  |  | **Crude** | | **Adjusted^a^** | |  | **Crude** | | **Adjusted^a^** | |
|  |  | **Relative Hazard (95% C.I.)** | **P value** | **Relative Hazard (95% C.I.)** | **P value** |  | **Relative Hazard (95% C.I.)** | **P value** | **Relative Hazard (95% C.I.)** | **P value** |
| **Age** |  | 0.99 (0.98-1.00) | 0.166 | 1.00 (0.98-1.01) | 0.498 |  | 1.01 (1.00-1.02) | 0.178 | 1.00 (0.99-1.02) | 0.563 |
| **Gender** |  | 1.29 (0.91-1.82) | 0.156 | 1.35 (0.88-2.07) | 0.169 |  | 0.91 (0.67-1.24) | 0.538 | 1.07 (0.74-1.55) | 0.702 |
| **Risk Factor:** |  |  |  |  |  |  |  |  |  |  |
| Homosexual^b^ |  | 1 |  | 1 |  |  | 1 |  | 1 |  |
| Heterosexual |  | 0.84 (0.61-1.17) | 0.302 | 0.91 (0.60-1.38) | 0.651 |  | **1.45 (1.08-1.95)** | **0.013** | **1.56 (1.08-2.25)** | **0.018** |
| Drug addiction |  | 0.63 (0.31-1.31) | 0.215 | 0.61 (0.26-1.45) | 0.261 |  | 0.88 (0.50-1.57) | 0.673 | 0.80 (0.40-1.62) | 0.540 |
| Sexual |  | 0.96 (0.62-1.50) | 0.868 | 0.86 (0.53-1.39) | 0.544 |  | 1.08 (0.71-1.63) | 0.721 | 0.86 (0.55-1.36) | 0.528 |
| Other or unknown |  | 0.79 (0.57-1.11) | 0.181 | 1.14 (0.76-1.71) | 0.513 |  | 0.91 (0.66-1.24) | 0.537 | 0.90 (0.62-1.31) | 0.577 |
| **Pre-HAART CD4 cell count (cells/mm^3^)**  ≤50^b^ |  | 1 |  | 1 |  |  | 1 |  | 1 |  |
| 51-100 |  | **1.80 (1.02-3.18)** | **0.043** | **2.24 (1.20-4.18)** | **0.011** |  | 0.75 (0.45-1.26) | 0.281 | **0.43 (0.24-0.76)** | **0.004** |
| 101-200 |  | **1.74 (1.09-2.77)** | **0.021** | **2.28 (1.36-3.83)** | **0.002** |  | 0.86 (0.57-1.32) | 0.499 | 0.64 (0.39-1.04) | 0.072 |
| 201-350 |  | **1.75 (1.17-2.61)** | **0.006** | **2.41 (1.49-3.88)** | **<0.001** |  | 1.22 (0.86-1.73) | 0.265 | 0.75 (0.49-1.15) | 0.188 |
| >350 |  | **1.67 (1.07-2.61)** | **0.024** | **2.21 (1.33-3.67)** | **0.002** |  | 1.14 (0.78-1.67) | 0.502 | 0.73 (0.46-0.13) | 0.158 |
| **Pre-HAART HIV-RNA (copies/mL)** |  |  |  |  |  |  |  |  |  |  |
| ≤30,000^b^ |  | 1 |  | 1 |  |  | 1 |  | 1 |  |
| 30,001-100,000 |  | 0.95 (0.65-1.38) | 0.778 | 1.05 (0.70-1.57) | 0.825 |  | 0.74 (0.53-1.04) | 0.083 | 0.81 (0.57-1.17) | 0.259 |
| 100,001-300,000 |  | 1.32 (0.93-1.86) | 0.117 | **1.62 (1.10-2.39)** | **0.015** |  | **0.62 (0.45-0.85)** | **0.003** | **0.61 (0.43-0.87)** | **0.007** |
| 300,001-500,000 |  | 0.92 (0.54-1.58) | 0.769 | 1.30 (0.72-2.34) | 0.390 |  | **0.56 (0.34-0.92)** | **0.022** | **0.44 (0.26-0.76)** | **0.003** |
| 500,001-1,000,000 |  | 1.10 (0.70-1.76) | 0.673 | 1.50 (0.87-2.58) | 0.142 |  | **0.38 (0.25-0.58)** | **<0.001** | **0.41 (0.25-0.67)** | **<0.001** |
| >1,000,000 |  | **1.72 (1.00-2.94)** | **0.048** | **2.82 (1.46-5.45)** | **0.002** |  | **0.39 (0.23-0.66)** | **<0.001** | **0.28 (0.15-0.52)** | **<0.001** |
| **Hepatitis C coinfetion** |  | 0.76 (0.46-1.26) | 0.290 | 0.94 (0.50-1.76) | 0.846 |  | 0.97 (0.62-1.50) | 0.880 | 1.39 (0.80-2.42) | 0.248 |
| **Year of starting treatment** |  | **1.12 (1.03-1.22)** | **0.009** | 1.11 (0.98-1.24) | 0.093 |  | 1.01 (0.93-1.09) | 0.885 | 0.95 (0.85-1.05) | 0.300 |
| **Transmitted Drug resistance** |  | 0.91 (0.57-1.46) | 0.696 | 1.02 (0.60-1.70) | 0.954 |  | 1.12 (0.72-1.73) | 0.620 | 1.06 (0.64-1.75) | 0.813 |
| **Third drug used** |  |  |  |  |  |  |  |  |  |  |
| NNRTI^b^ |  | 1 |  | 1 |  |  | 1 |  | 1 |  |
| Boosted PI |  | 0.92 (0.70-1.22) | 0.566 | 1.00 (0.73-1.36) | 0.995 |  | 0.87 (0.68-1.12) | 0.279 | 1.08 (0.81-1.42) | 0.604 |
| Raltegravir |  | 1.08 (0.70-1.67) | 0.732 | 0.78 (0.41-1.48) | 0.441 |  | **2.21 (1.47-3.33)** | **<0.001** | **5.03 (2.85-8.86)** | **<0.001** |
| **NRTI backbone used** |  |  |  |  |  |  |  |  |  |  |
| TDF+FTC^b^ |  | 1 |  | 1 |  |  | 1 |  | 1 |  |
| AZT+3TC |  | **0.46 (0.25-0.85)** | **0.013** | **0.45 (0.21-0.95)** | **0.036** |  | 0.98 (0.60-1.60) | 0.931 | 0.79 (0.43-1.45) | 0.455 |
| Others |  | 0.92 (0.60-1.39) | 0.681 | 0.97 (0.55-1.72) | 0.927 |  | 1.33 (0.89-1.99) | 0.160 | 1.02 (0.64-1.62) | 0.946 |
| **>3 drugs used** |  | **1.63 (1.02-2.62)** | **0.041** | **1.95 (1.06-3.59)** | **0.032** |  | 1.14 (0.72-1.82) | 0.580 | 0.73 (0.38-1.39) | 0.332 |
| a: Adjusted for: genotypically-inferred tropism, age, gender, risk factor, pre-HAART CD4 cell count, pre-HAART HIV-RNA, hepatitis C coinfection, transmitted drug resistance, year of starting treatment, third drug used (non-nucleoside reverse transcriptase inhibitor vs. boosted protease inhibitor vs. raltegravir), NRTI backbone used (tenofovir + emtricitabine vs. zidovudine + lamivudine vs. others), number of drug administered (≤3 vs. >3 drugs). b: Reference group (dummy). AZT: zidovudine. FTC: emtricitabine. NRTI: Nucleos(t)ide reverse transcriptase inhibitor. NNRTI: Non-NRTI. PI: protease inhibitor. TDF: tenofovir. 3TC: lamivudine. Boldface indicates the factors that were significantly associated (p<0.05) with viro-immunological response. | | | | | | | | | | |
